# Cross-Sectional Investigation of Brain Volume in Dyslexia

**DOI:** 10.3389/fneur.2022.847919

**Published:** 2022-03-08

**Authors:** Carolin Ligges, Marc Ligges, Christian Gaser

**Affiliations:** ^1^Department of Child and Adolescent Psychiatry, Psychosomatic Medicine and Psychotherapy, Jena University Hospital, Jena, Germany; ^2^Department of Psychiatry and Psychotherapy, Jena University Hospital, Jena, Germany; ^3^Department of Neurology, Jena University Hospital, Jena, Germany

**Keywords:** reading, developmental dyslexia, local brain volume, gray matter, VBM, structural MRI

## Abstract

The goal of the study was to determine whether dyslexia is associated with differences in local brain volume, and whether these local brain volume differences show cross-sectional age-effects. We investigated the local volume of gray and white brain matter with voxel-based morphometry (VBM) as well as reading performance in three age groups of dyslexic and neurotypical normal reading subjects (children, teenagers and adults). Performance data demonstrate a steady improvement of reading skills in both neurotypical as well as dyslexic readers. However, the pattern of gray matter volumes tell a different story: the children are the only group with significant differences between neurotypical and dyslexic readers in local gray matter brain volume. These differences are localized in brain areas associated with the reading network (angular, middle temporal and inferior temporal gyrus as well as the cerebellum). Yet the comparison of neurotypical and normal readers over the age groups shows that the steady increase in performance in neurotypical readers is accompanied by a steady decrease of gray matter volume, whereas the brain volumes of dyslexic readers do not show this linear correlation between brain volume and performance. This is further evidence that dyslexia is a disorder with a neuroanatomical basis in the form of a lower volume of gray matter in parts of the reading network in early dyslexic readers. The present data point out that network shaping processes in gray matter volume in the reading network does take place over age in dyslexia. Yet this neural foundation does not seem to be sufficient to allow normal reading performances even in adults with dyslexia. Thus dyslexia is a disorder with lifelong consequences, which is why consistent support for affected individuals in their educational and professional careers is of great importance. Longitudinal studies are needed to verify whether this holds as a valid pattern or whether there is evidence of greater interindividual variance in the neuroanatomy of dyslexia.

## Introduction

Developmental dyslexia affects about 5 percent of the population depending on diagnostic criteria (1). Even nowadays affected individuals still struggle to receive adequate support, thus dyslexia has far-reaching consequences for the suffering individual on scholastic, psychological and socio-economic levels (2–4). Individuals affected by dyslexia have problems with fluent and/or accurate reading, spelling and the proper acquisition of grapheme-phoneme correspondence. Dyslexia is not caused by a general cognitive impairment or a lack of an opportunity to learn (5). Several cognitive, sensoric and neurobiological deficits are suggested to cause dyslexia (6) which in turn are supposed to impact the acquisition and automation of the reading and spelling process (7–10). Reading processes take place in a large neural reading network comprised of broad areas in the dorsal superior temporal, ventral inferior temporal, and inferior frontal brain [e.g., (11–13)]. The extent of the involvement of this particular reading network depends on cognitive demands of the reading task (14). Phonological processing strongly involves the dorsal reading system, whereas the ventral system is more involved with visual word form processing as well as the transfer of letter shape to phonological content (transfer of visual input to linguistic output units) (15). The extent to which either the dorsal or ventral system is most involved depends on the skill level involved in the reading process: beginning readers rely more on the phonological dorsal system and skilled readers rely more on the well-trained ventral visual reading system (16–18). In those suffering from dyslexia, the dorsal and inferior frontal components of the neural reading network consistently display functional differences compared to the neural reading network of neurotypical readers (19–21).

Voxel-based morphometry (VBM) is used in neuroimaging studies to determine whether dyslexia is associated with differences in local gray and white brain matter volumes in the reading network. VBM, without using a region of interest approach, is a fully automated method to identify regions of local volume differences in the whole brain.

Studies investigating the differences in local brain matter volume between neurotypical reading controls and readers with dyslexia identify various areas within the oral and written language networks [for an overview see (22–28)]. Regions which repeatedly show differences in local brain volume between neurotypical reading and dyslexic subjects are in inferior parietal (29), temporo-parietal (29, 30) and superior temporal regions (31), the inferior frontal gyrus, the left and right fusiform gyrus (32) and the cerebellum (29, 32).

Recent studies using VBM demonstrate variations in brain matter volume in prereading children at risk for dyslexia (33–35), across different language systems (36, 37) as well as differences in dyslexic children after a reading intervention (38). VBM studies thus indicate that dyslexia-specific morphometric differences can already be observed before the acquisition of written language and that these differences can be referred to as early neuroanatomical signatures for the subsequent reading problems. Finding no differences in dyslexia-specific VBM profiles across different language systems, Silani et al. (36) assume that there could be a common neuroanatomical basis irrespective of the language system. VBM studies also highlight the flexibility of the human brain since these studies demonstrate that these differences can be influenced by reading experience or training.

Several studies on the association between functional and volumetric differences in dyslexia detect that functional and volumetric differences between neurotypical and dyslexic readers are not only coexisting results, but can also serve as indicators of associated disorder characteristics. As these studies demonstrate (30, 39) there is evidence that areas which show dyslexia-specific hypo-activations in functional neuroimaging studies are related to differences in brain matter volumes between neurotypical and dyslexic readers.

Methodological inconsistencies between existing VBM studies on dyslexia make it hard to compare results since they are strongly influenced by the language system (shallow vs. deep orthographic system), experimental designs (subject samples, diagnostic criteria for dyslexia) as well as methods for data acquisition and analysis, [i.e., modulation for absolute GMV, registration algorithms using either a group-specific or a priori template, kernel size used for smoothing as well as differences on the level of statistical analysis (i.e., level of statistical correction for multiple tests)]. These inconsistencies can lead to differences in the findings of the studies (22, 37). The investigation of age dependent differences in brain volume of dyslexic readers by comparing different study-results is thus difficult.

To our knowledge, no study has yet addressed characteristics in brain matter volume with VBM in dyslexic readers over a large age range.

The aim of the present study is therefore to investigate age-dependent differences in local brain matter volume in developmental dyslexia. We hypothesize that dyslexic and neurotypical readers show different local brain volumes in areas related to the neuronal reading network and that these local brain volume differences show cross-sectional age-effects when comparing three groups (children, teenagers and adults with dyslexia compared to neurotypical normal reading, age-matched controls). Since we apply the same experimental design as well as the same parameters for data acquisition and analysis to all groups, we should be able to overcome the methodological pitfalls mentioned above.

## Materials and Methods

### Subjects

The study comprises three groups of subjects with dyslexia (children, teenagers and adults) as well as three groups of neurotypical normal reading subjects matched a posteriori according to age and nonverbal IQ (children, teenagers and adults).

A total of 21 neurotypical children, 24 neurotypical teenagers, 27 neurotypical adults, 22 dyslexic children, 18 dyslexic teenagers as well as 22 dyslexic adults took part in the diagnostic session and MRI data acquisition. Due to inferior MRI Data quality of some participants, these individuals were excluded from the analysis. Thus, the analyses in the present study are based on a final sample of 20 neurotypical children, 21 neurotypical teenagers, 26 neurotypical adults, 21 dyslexic children, 17 dyslexic teenagers as well as 20 dyslexic adults.

The study was approved by the local Ethics Committee at the Jena University Hospital. Subjects and legal guardians were informed verbally and with written materials about the experimental procedure. All individual participants included in the study and their legal guardians gave written informed consent for their participation. They were informed that all published data are fully anonymized. All procedures performed in this study are in accordance with the ethical standards of the institutional and/or national research committee and with the 1964 Helsinki declaration and its later amendments or comparable ethical standards.

### Assessment of Sample Criteria

During a diagnostic session several standardized tests were applied. These data were used to assess the sample criteria. The IQ of all subjects had to be ≥85. Sample criteria for dyslexia was a double discrepancy: ≥1.5 standard deviations between nonverbal IQ and reading and spelling performance as well as reading and spelling performance < percentile rank 15. Sample criteria for neurotypical controls was discrepancy between nonverbal IQ and reading as well as spelling performance < 1 standard deviation.

Subjects with uncorrected impairments of sight or hearing, bilingual education, neurological or psychiatric disorders (especially ADHD) were excluded based on the information obtained in a detailed clinical screening interview as well as performance in an attention test. All subjects were right handed according to verbal assessment. For an overview of the results regarding study criteria please refer to Table 1.

**Table 1:** Sample criteria.

	**NT-child *n* = 20**	**D-child *n* = 21**	**NT-teen *n* = 21**	**D-teen *n* = 17**	**NT-adult *n* = 26**	**D-adult *n* = 20**	
	***M (SD)***	***M (SD)***	***M (SD)***	***M (SD)***	***M (SD)***	***M (SD)***	**ANOVA statistics**
Age (year, month)	10.60 (0.94)	10.38 (0.92)	13.31 (1.37)	13.92 (1.50)	26.25 (5.29)	26.20 (9.54)	ME read-lev: F (1.125) = 0.02; n.s.; ME age: F (2.125) = 140.97; *p* <0.001; inter F (2.125) = 0.09, n.s.
IQ	109.15 (15.52)	108.14 (15.13)	102.97 (12.81)	100.76 (14.61)	106.89 (16.17)	100.90 (12.15)	ME read-lev: F (1.125) = 1.37; n.s.; ME age: F (2.125) = 2.28; n.s.; inter F (2.125) = 0.35, n.s.
Spelling (IQ-scale)	105.34 (11.96)	69.84 (8.45)	102.05 (10.64)	66.18 (13.65)	104.85 (13.67)	63.64 (11.24)	ME read-lev: F (1.125) = 313.07; *p* <0.001; ME age: F (2.125) = 1.15; n.s.; inter F (2.125) = 0.79, n.s.
Reading (IQ-scale)	101.68 (7.22)	74.20 (8.15)	106.19 (5.68)	83.50 (13.06)	110.77 (3.02)	99.64 (8.72)	ME read-lev: F (1.125) = 207.52; *p* <0.001; ME age: F (2.125) = 52.71; *p* <0.001; inter F (2.125) = 12.36, *p* <0.001

*NT-child, neurotypical reading children; D-child, children with dyslexia; NT-teen, neurotypical reading teenagers; D-teen, teenagers with dyslexia; NT-adult, neurotypical reading adults; D-adult, adults with dyslexia; n, sample-size of subgroup; sd, standard deviation; IQ, intelligence quotient; ME, main effect; age, children, teenagers, adults; read-lev, neurotypical readers and readers with dyslexia; inter, interaction; %, percent; age, year, month; spelling and reading scores, transformed to IQ-scale for better comparison*.

As a measure of nonverbal intelligence, Raven's Standard Progressive Matrices was administered (40). The test measures the subjects' reasoning ability, the educative (“meaning-making”) component of Spearman's g. It is comprised of multiple-choice questions. For each test item, the subject is asked to identify the missing element that completes a pattern. Reading fluency and accuracy both in single word as well as in text reading was assessed by means of a standardized reading test [Zürcher Lesetest, (41)]. In this test subjects read different lists of single words as well as different texts respective to class level. Since the ZLT can be applied to a wide age range, the use of various test procedures was not necessary. The test acquires scores for the time needed as well as the errors made while reading the target words and texts. Spelling performance was assessed by dictation of a gap text by means of standardized spelling tests appropriate for the respective class or age level (42–44). The tests acquire the number of misspelled words.

Attention was assessed via the so-called “cross-out-test.” In this test, the subject is confronted with visually similar items and is asked to cross out certain target items. Attention as well as the tendency toward impulsive behavior is assessed via the speed and accuracy with which differentiation between the visually similar items is achieved by the subject. This test was administered in order to ensure that deficits in attention span do not confound the results [d2, (45)].

### Reading Experiment: Assessment of Reading and Phonological Skills as Dependent Variables

Since phonological processing plays a crucial role in the reading process, especially for beginning readers, we acquired additional neuropsychological data on reading skill and phonological decoding skills using single word reading (e.g., Baum Bein), reading of pseudowords (e.g., Bilza Bilaz) as well as the rhyming of pseudowords (e.g., Jurde Surde).

We used these tasks in order to trigger phonological reading processes at different levels of difficulty, requiring different levels of phonological skill: Reading of frequently used regular single words should require lowermost phonological skills. These words should engage highly automatized whole word reading strategies. Pseudoword reading should exercise an increased demand for phonological processing, since the pseudowords do not exist in the common vocabulary and have no entry in the mental lexicon. The unknown word material must be read by using phonological grapheme-phoneme correspondence skills. Rhyming of pseudowords is thought to require the most phonological processing skill as, in addition to the grapheme-phoneme correspondence skill, phonological short time storage is needed to keep up the phonological code of the pseudoword in order to make the rhyme judgment (10).

For single word reading, frequently used German words were taken from third grade vocabulary (46, 47). Pseudowords were created on the basis of real words in which first the vowels were exchanged followed by stepwise exchange of consonants, until there was no longer an association for an existing German word. Subjects decided whether two items that were visually presented side by side on the computer screen (i.e., Baum Bein) were identical or not. Each decision required a key press, so that responses were registered through a key press of either index (stimuli are the same) or middle finger (stimuli are not the same) of the right hand. Reaction time and error rate were acquired via ERTS (48). The presentation rate of the stimuli was not fixed but subject-controlled with a maximal stimulus-presentation time of 5 s. If the subject pressed a key within these 5 s, the next pair of stimuli was presented after an inter-trial interval of 500 ms. The computer automatically switched to the next trial if the reaction time of 5 s was exceeded.

**Figure 1:**
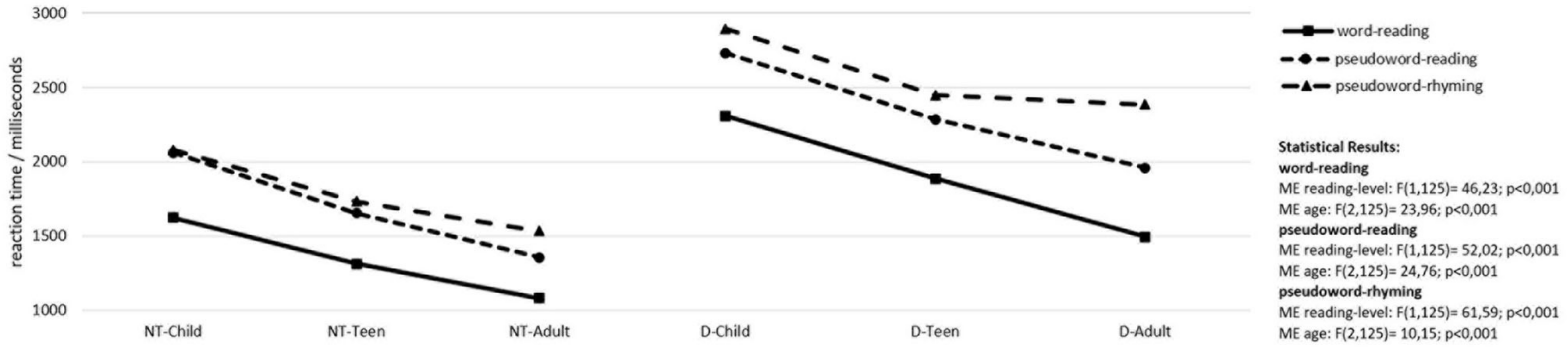
Word reading and phonological processing skills. NT-child, neurotypical reading children; D-child, children with dyslexia; NT-teen, neurotypical reading teenagers; D-teen, teenagers with dyslexia; NT-adult, neurotypical reading adults; D-adult, adults with dyslexia; ME, main effect; age, children, teenagers, adults; reading-level, neurotypical readers, readers with dyslexia. Only significant results are reported.

We hypothesized that improvement in reading and phonological skills (lower reaction times and error rates) in neurotypical and dyslexic readers should be observed from children to teenagers to adults and a potential phonological processing deficit should be reflected by increased reaction times and error rates over the three tasks in dyslexics compared to neurotypical readers. Performance in word reading, pseudoword reading and pseudoword rhyming is depicted in Figure 1.

### MRI Data Acquisition

MRI data were acquired using a Siemens Magnetom Vision 1.5 Tesla MRI Scanner (Erlangen, Germany). The head was fixated inside the head coil with extra padding in order to avoid movement artifacts. A high-resolution anatomical dataset of the whole brain (192 slices, T1-weighted, TR = 15 ms, TE = 5 ms, flip angle 30°, 1 mm slice thickness, magnetization prepared rapid acquisition gradient echo sequence) was acquired.

### Data Analysis / Diagnostic Data

Diagnostic data as well as the performance data on word reading and phonological processing were analyzed using 3 x 2 ANOVA with the factor “age group” (children, teenagers and adults) and the factor “reading-level” (neurotypical readers vs. readers with dyslexia).

### Data Analysis / Voxel-Based Morphometry

Data Pre-processing and analysis were performed using Statistical Parametric Mapping software (Institute of Neurology, London, UK, http://www.fil.ion.ucl.ac.uk/spm/software/spm8, SPM, RRID:SCR_007037, version 8). For morphometric analysis of the data, we used voxel-based morphometry (VBM). This method involves the following steps: (1) spatial normalization of all images to a standardized anatomical space by removing differences in overall size, position, and global shape; (2) extracting gray and white matter from the normalized images; and (3) analyzing differences in local gray and white matter values across the whole brain (49). We applied an optimized method of VBM (50) using the VBM8 Toolbox (http://dbm.neuro.uni-jena.de/vbm; VBM toolbox, RRID:SCR_014196, version 8) for both gray and white matter.

The segmentation procedure is further refined by accounting for partial volume effects (51), by applying adaptive maximum a posteriori estimations (52), and by applying a hidden Markov random field model (53). Because spatial normalization expands and contracts for some brain regions we scaled the segmented images by the amount of contraction, so that the total amount of gray or white matter in the images remained the same as it would be in the original images.

Due to the large differences in gray matter brain volume between the groups of children, teenagers, and adults we created a sample-specific template for spatial normalization. An iterative high-dimensional normalization approach provided by the DARTEL toolbox (54) was applied to the segmented tissue maps in order to normalize all images to a template. The DARTEL algorithm started with low-dimensional spatial normalization and calculated the average of all normalized segmentations. This averaged image was used in the next iteration and spatial resolution of the normalization was enhanced. This iteration scheme was repeated while the dimensionality of spatial normalization was increased. The result was a high-dimensionally warped brain with minimal bias, because a sample-specific template was used.

The resulting gray and white matter images were finally smoothed with a Gaussian kernel of 8 mm FWHM. We restricted the statistical analysis to areas with a minimum probability value of 0.1 to avoid possible edge-effects around tissue borders. Differences in local gray and white matter volume across the whole brain are analyzed with voxel-by-voxel *t*-test using the general linear model. We use a 3 × 2 ANOVA with factors age group (children, teenager and adults) and reading-level (neurotypical readers and readers with dyslexia) to test for differences between each group with dyslexia and their neurotypical control group. Since we use a modulation for Non-linear effects only that considers overall brain size, there is no need to correct for total intracranial volume (TIV) in the statistical model.

Furthermore, we tested for an interaction of the factors age group and reading-level. Results were considered significant for *p* < 0.05, corrected for multiple comparisons using FWE based on threshold-free cluster enhancement (TFCE), which avoids using an arbitrary threshold for the initial cluster-formation (55). Corresponding coordinates for each significant region are reported in Montreal Neurological Institute (MNI) space.

Additionally we performed correlation analyses between reading performance and gray matter volumes as well as age and gray matter volumes of those gray matter clusters which showed significant main effects for reading level in the comparison of neurotypical and dyslexic readers. In clusters with two cluster maxima, we chose the cluster with the larger TFCE values for the correlations (Cluster 1: MNI coordinates −51 −63 25; Cluster 3: MNI coordinates −42 −67 −29; Cluster 5: 47 −60 −32).

## Results

### Sample Criteria

For a summary of the descriptive statistics as well as the statistical results of the 3 x 2 ANOVAs of the sample criteria data please refer to Table 1. Reading and spelling performance of the neurotypical readers stays within the normal range for all three age groups, whereas reading performance shows better performance with rising age for the dyslexic readers, reaching normal reading performance levels in adulthood. However spelling performance remains below average for all three age groups of dyslexic readers.

### Reading and Phonological Skills

For performances in word and pseudoword reading and pseudoword rhyming there are highly significant main effects for factors age-group and reading-level. For reading of words and pseudowords there is a steady increase in performance from children to teenagers to adults in the neurotypical as well as in the dyslexic readers even though the latter are consistently slower over all three age groups compared to the neurotypical readers. For rhyming of pseudowords (the task with the highest demands on phonological processing skills), there is also an improved performance from children to teenagers in the neurotypical readers and the readers with dyslexia. However, adults with dyslexia do not show any further increase in performance. Their performance remains at the level of the teenagers with dyslexia.

### Voxel-Based Morphometry

#### Gray Matter

**Figure 2:**
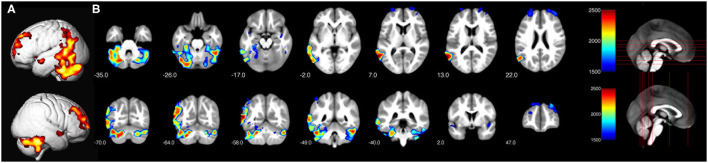
Significant differences in local gray matter (TFCE-statistic, FWE corrected for multiple comparisons, *p* ≤ 0.05) for neurotypical reading children > children with dyslexia. **(A)** Render view of all clusters. **(B)** Depiction of axial and coronal slices in order to illustrate the displayed slices.

Only children show significant main effects for the factor reading-level regarding local gray matter volume differences between neurotypical readers vs. readers with dyslexia (on a level corrected for multiple statistical comparisons, TFCE-statistic, FWE corrected, *p* = 0.05). Neurotypical children show significantly larger local volumes of gray matter than children with dyslexia in large left-sided temporo-parietal and frontal clusters [encompassing the inferior temporal gyrus, fusiform gyrus, middle temporal gyrus, V5, middle occipital gyrus, superior temporal gyrus, inferior parietal gyrus, angular gyrus, supramarginal gyrus, left and right superior temporal gyrus (encompassing the insulae), left and right cerebellum as well as left and right prefrontal clusters (encompassing the superior, middle and medial frontal gyrus)]. See Figure 2 for the differences in gray matter volume of the children and Table 2 for the coordinates of the local cluster maxima.

**Table 2:** Coordinates and anatomical regions of main effect neurotypical reading children > children with dyslexia.

**Cluster size**	***p* (FWE-cor)**	**TFCE**	***x***	***y***	***z***	**Hemisphere**	**AAL region**
**22,069**	**0.012**	**2521.43**	**−63**	**−60**	**11**	**Left**	**Middle temporal, angular**
	0.017	2382.85	−23	−68	−38	Left	Cerebellum
	0.018	2342.32	−23	−60	−32	Left	Cerebellum
**8,679**	**0.017**	**2353.06**	**50**	**−50**	**−29**	**Left**	**Inferior temporal, cerebellum, fusiform**
	0.019	2273.18	47	−62	−32	Right	Cerebellum, inferior temporal
	0.020	2204.81	41	−72	−32	Right	Cerebellum
**3,610**	**0.029**	**1830.28**	**30**	**45**	**44**	**Right**	**Superior and middle frontal**
	0.029	1813.50	24	39	45	Right	Superior and middle frontal
	0.035	1709.09	42	53	26	Right	Inferior frontal, middle and superior frontal
**3,446**	**0.036**	**1678.57**	**−27**	**42**	**47**	**Left**	**Superior and middle frontal**
	0.036	1656.90	−5	53	45	Left	Superior frontal
	0.036	1656.17	−17	51	45	Left	Superior frontal
**570**	**0.039**	**1595.57**	**45**	**3**	**−12**	**Right**	**Superior temporal, insula**
	0.040	1580.81	41	−9	−18	Right	Hippocampus, superior temporal, fusiform
**446**	**0.042**	**1540.42**	**−51**	**0**	**−12**	**Left**	**Superior and middle temporal**
	0.043	1528.52	−42	5	−15	Left	Superior temporal, insula
**209**	**0.044**	**1509.94**	**−50**	**−45**	**20**	**Left**	**Superior and middle temporal, supramarginal**
	0.046	1486.35	−42	−36	33	Left	Supramarginal, inferior parietal, postcentral
	0.047	1477.07	−44	−47	29	Left	Supramarginal, angular, superior temporal
**152**	**0.048**	**1468.62**	**9**	**−54**	**29**	**Right**	**Precuneus, posterior and middle cingulum**
	0.048	1459.51	11	−47	26	Right	Posterior and middle cingulum, precuneus
**93**	**0.048**	**1468.55**	**−29**	**15**	**−3**	**Left**	**Insula, putamen, inferior frontal**
**23**	**0.048**	**1457.90**	**−9**	**−81**	**−8**	**Left**	**Lingual, calcerinus, cerebellum**
**16**	**0.048**	**1457.70**	**−33**	**53**	**3**	**Left**	**Middle and superior frontal**

*p, level of significance, only significant results corrected for multiple comparisons (FWE) are reported; TFCE, Threshold Free Cluster Enhancement; AAL, Automated Anatomical Labeling. Values in bold denote the respective local cluster maxima*.

Three clusters, which are part of the left-sided reading network (angular gyrus and middle temporal gyrus), the left and right cerebellum, and the right fusiform gyrus show significant interaction effects of the factors age-group and reading-level regarding local gray matter brain volume.

**Figure 3:**
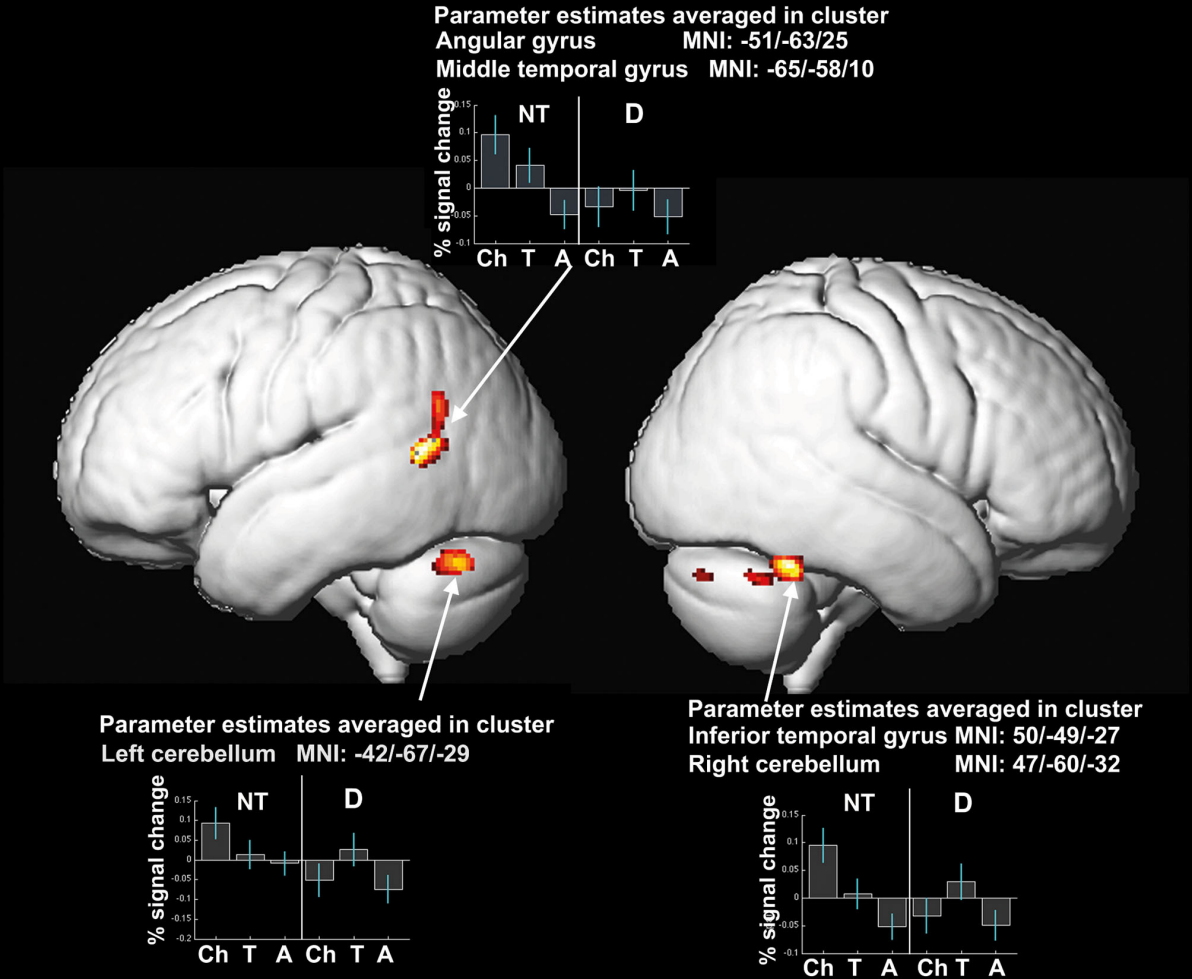
Significant differences in local gray matter (uncorrected, *p* ≤ 0.001, expected cluster level according to expected numbers of voxel per cluster: 75 voxel) as well as % signal changes in the cluster centers for interaction effect of factors age-group and reading-level. Coordinates of the clusters and percent signal change of the averaged clusters are depicted. NT, neurotypical readers; D, readers with dyslexia; Ch, children; T, teenagers; A, adults.

The percent signal change in the cluster maxima as depicted in Figure 3 demonstrates, that the groups of readers with dyslexia (see Figure 3) mainly drive this interaction effect. Neurotypical readers present a steady decrease of percent signal change in all clusters from children to teenagers to adults. Whereas the comparison of the percent signal change in the readers with dyslexia displays an inverted U-shape: comparing the groups with dyslexia, children show smaller volumes than teenagers, and for teenagers larger volumes can be detected than for adults. Adults on the other hand show the smallest local volumes of gray matter compared to children and teenagers (see Figure 3).

#### Correlation Analysis

**Figure 4:**
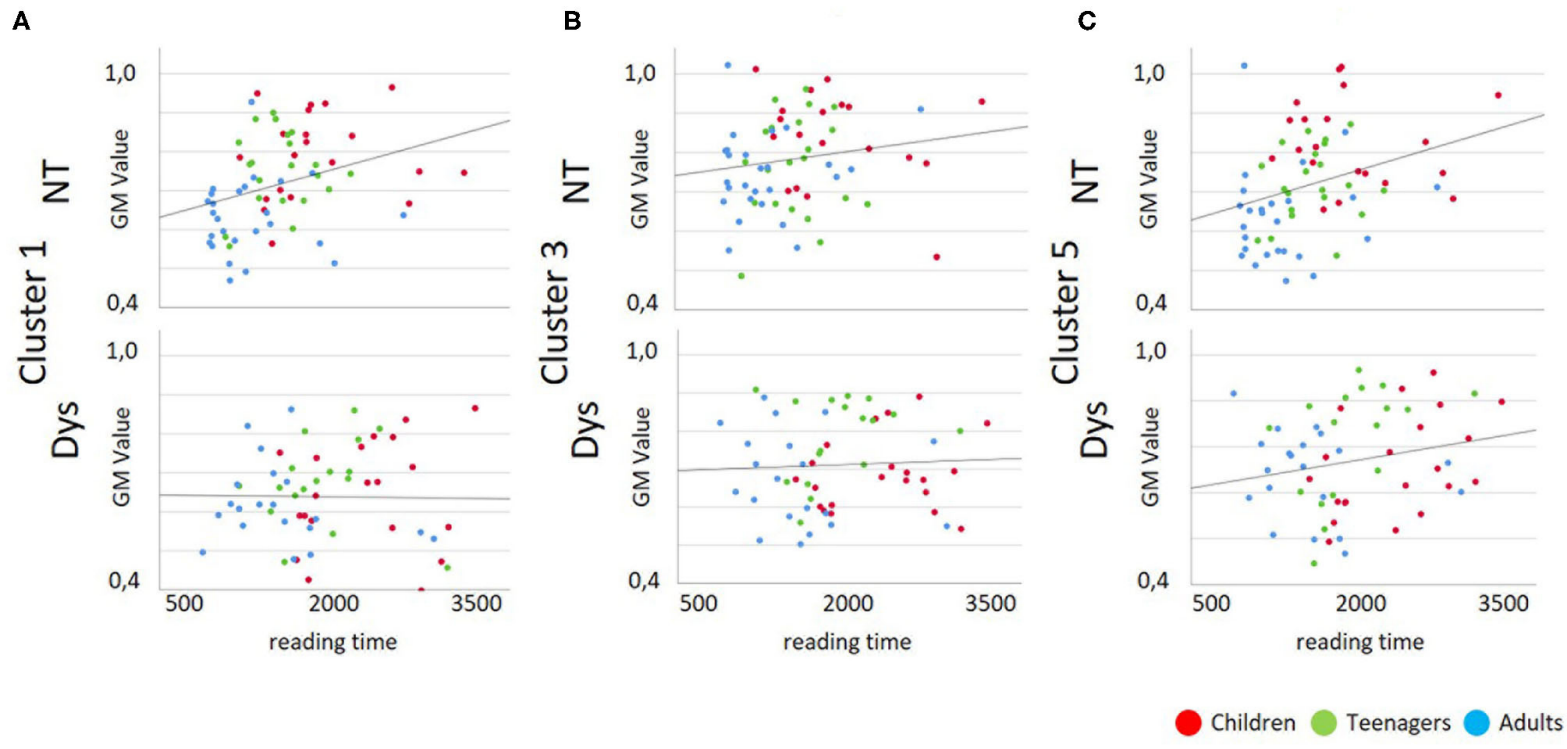
Scatterplots for Gray Matter Volume vs. Reading Performance; Dys, Dyslexic Readers; NT, Neurotypical Readers; GM, Gray Matter Volume; reading time in milliseconds. **(A)** Cluster 1/MNI-coordinates: −51/−63/25, **(B)** Cluster 3/MNI-coordinates: −42/−67/−29, **(C)** Cluster 5/MNI-coordinates: 47/−60/−32.

**Figure 5:**
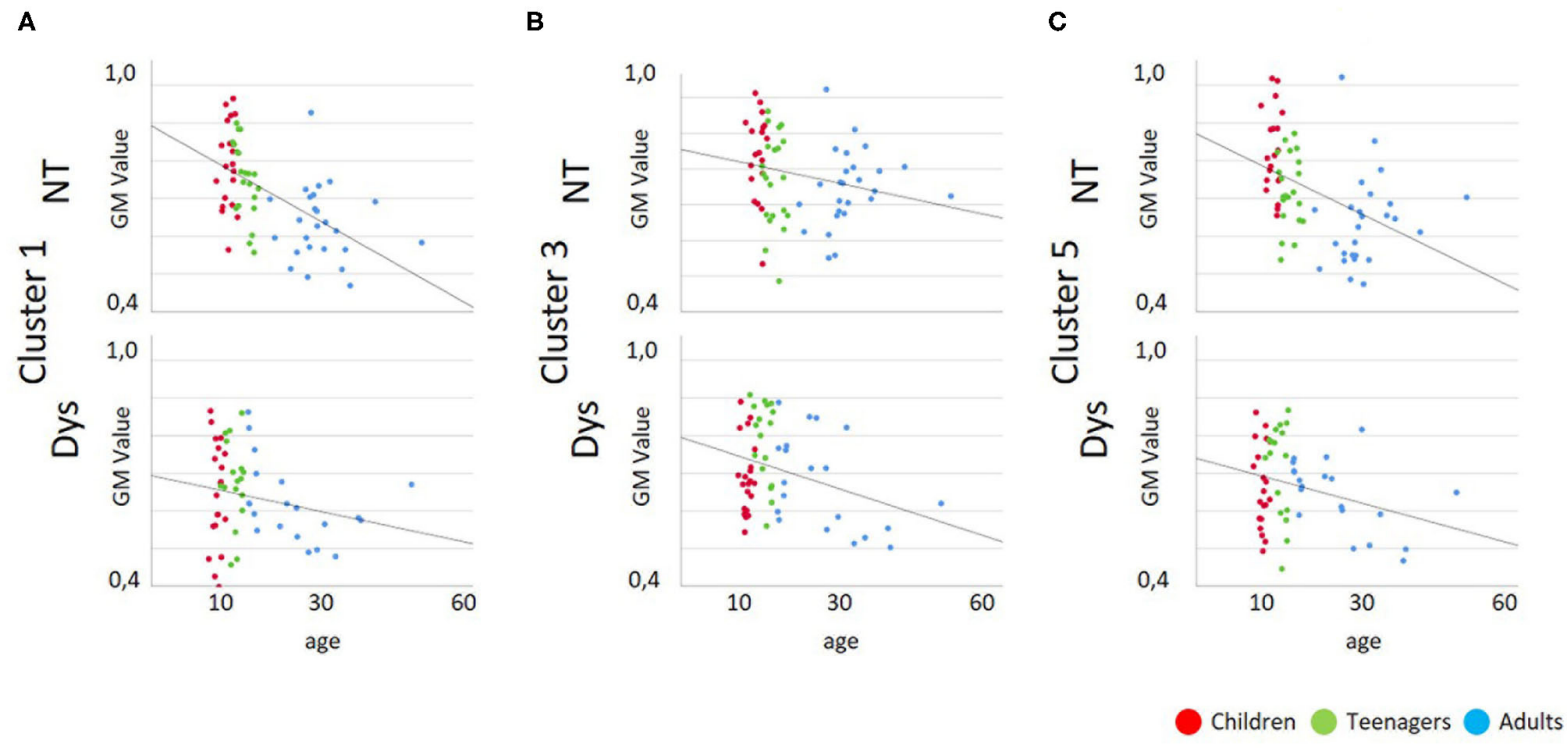
Scatterplots for Gray Matter Volume vs. Age; Dys, Dyslexic Readers; NT, Neurotypical Readers; GM, Gray Matter Volume; age in years. **(A)** Cluster 1/MNI-coordinates: −51/−63/25, **(B)** Cluster 3/MNI-coordinates: −42/−67/−29, **(C)** Cluster 5/MNI-coordinates: 47/−60/−32.

As depicted in Tables 3 and 4 the correlation analysis between reading performance and gray matter volume shows two significant correlations for neurotypical readers in Cluster 1 and 5, there are no significant correlations between performance and gray matter volume for dyslexic readers. Looking at correlations between age and gray matter volume, there are significant negative correlations for neurotypical readers in Cluster 1 and 5 and significant negative correlations for dyslexic readers in Cluster 3 and 5. Scatterplots for these correlations are depicted in Figures 4, 5.

**Table 3:** Correlational analysis reading performance and gray matter volume.

	**Cluster 1**		**Cluster 3**		**Cluster 5**	
	***r***	***p***	***r***	***p***	***r***	***p***
Neurotypical readers	0.319	0.008	0.154	0.212	0.315	0.010
Dyslexic readers	−0.015	0.912	0.052	0.699	0.213	0.108

*r, correlation coefficient; p, level of significance; Cluster 1, MNI coordinates −51 −63 25; Cluster 3, MNI coordinates −42 −67 −29; Cluster 5, 47 −60 −32*.

**Table 4:** Correlational analysis age and gray matter volume.

	**Cluster 1**		**Cluster 3**		**Cluster 5**	
	***r***	***p***	***r***	***p***	***r***	***p***
Neurotypical readers	−0.557	0.000	−0.215	0.080	−0.439	0.000
Dyslexic readers	−0.194	0.145	−0.317	0.015	−0.280	0.033

*r, correlation coefficient; p, level of significance; Cluster 1, MNI coordinates −51 −63 25; Cluster 3, MNI coordinates −42 −67 −29; Cluster 5, 47 −60 −32*.

#### White Matter

There are no significant differences in local white matter volume between the children, teenagers, and adults.

## Discussion

Reading and spelling performance of the dyslexic readers shows a typical clinical pattern across the different age groups: as proven by significant main effects for age-group, reading-level and interaction effects the reading performance in the dyslexic readers improves significantly over age, reaching average performance in adulthood, whereas spelling performance in dyslexic readers stays below average in all three age groups. The fact that the three groups of dyslexic readers show slight differences in spelling performance can probably be explained by the fact that different tests appropriate to their respective ages had to be used.

There are three main study results for the VBM data: (1) Only children display a main effect for the factor “reading-level” regarding differences in local gray matter volume. (2) The significant interaction effect for the factors “age-group” and “reading-level” can be explained by a significant correlation of performance and gray matter volume in neurotypical readers, showing that performance increase is accompanied by gray matter volume decrease over age. Dyslexic readers do not show these correlations between performance and gray matter volume. (3) There are no significant differences in white matter.

### Main Effect for Factor “Reading-Level” in the Children

Children show differences in local gray matter brain volume in temporo-parietal areas [fusiform gyrus, angular gyrus, supramarginal gyrus and superior temporal gyrus (insulae)]. These clusters correspond to findings from other VBM studies in which readers with dyslexia display anatomical differences in these particular brain areas (29–32). These areas are critical parts of the reading network since they are related to phonological processing [superior temporal and temporo-parietal brain areas, (56)], skilled and automated reading, visual-auditive integration and memory for word images [inferior temporal brain areas (15, 18)].

### Could This Main Effect Be Due to a Lack of Reading Experience?

Krafnick and colleagues (57) argue that differences in brain volume between neurotypical reading children and children with dyslexia are most likely due to a lack of reading experience, since the comparison to reading-level matched neurotypical children does not display a significant difference in volume. It is likely that the reading experience plays an important role in the shaping of the reading network, however differences in brain volume in prereading children with or without risk for dyslexia can also be observed (33, 35). Thus, the effects of the present study do not seem to be only due to a lack of reading experience.

In addition to the differences in gray matter volume observed in the temporo-parietal cluster, the present study also demonstrates differences in bilateral prefrontal regions. Black and colleagues (34) report reduced gray matter brain volume in the prefrontal brain areas of children at risk for dyslexia. They associate these reduced volumes with differences in functions such as naming, verbal fluency (58, 59), executive processing (2) and working memory. The working memory is an important factor in the development of efficient reading skills as this is the means by which speech sounds are sustained during reading. These anatomical differences in brain areas important for the development of automated reading skills, efficient phonological processing skills and verbal working memory could be the neuroanatomical correlate for a markedly poorer baseline in the development of reading and spelling skills.

### Association Between Performance and Brain Volume in Neurotypical Readers

The regular pattern of brain matter volume development over the whole life span is that of an inverted U-shape (60). Normally there is an increase of brain volume with a peak of brain volume around the age of 6–8 years, followed by a steady decrease of brain volume as an ongoing lifelong process.

The mechanism behind this phenomenon is called synaptic pruning (13, 61–63). In general it is assumed, that the key to better cognitive performance lies in neural processes like pruning and cortical thinning in order to create a well-functioning cortical network. We know from the neurotypical developing brain, that overproduction of neurons and connections are the starting point in brain development. The initial network is unorganized with many superfluous connections making communication disorganized and inefficient. It is suggested that the subsequent decrease in gray matter brain volume over age in the neurotypical brain reflects the sculpting process for a well-functioning mature brain with efficient neuronal networks (60).

Recent research confirms that experience-dependent changes in brain structures extends throughout the lifespan (13, 26, 64), and that reading experience in the neurotypical reading brain successively leads to a reduction of brain volume. There are also studies on reading development which demonstrate that lower brain volumes correspond to better reading skills (13, 64).

Performance data of the present study indicate that there is a steady increase in reading skill for all reading tasks in neurotypical readers from children, to the teenage years, to adulthood. This improvement in performance is accompanied by a steady decrease of gray matter volume. Thus, findings from our neurotypical reading control group agree with the literature insofar that better reading and spelling skills are related to lower volumes of gray matter brain volume in the reading network.

### Dyslexia Specific Differences in Brain Matter Volume

Compared to the brain volumes of the neurotypical readers in our sample, the dyslexic readers do not show these linear effects when looking at correlations of performance and brain volumes. The behavioral data indicate that neurotypical readers as well as dyslexic subjects show progress in reading and spelling performance with age. However, as correlational analyses results demonstrate, this steady increase in performance is not paralleled by a linear decrease of brain volume in dyslexic readers. Children with dyslexia demonstrate smaller gray matter brain volume and lower reading performance compared to the teenagers with dyslexia as can be seen in Figure 3. The improvement in reading skills in the teenagers with dyslexia is combined with larger brain volume in this group. This is not in line with the amount of brain volumes of the neurotypical reading control group in which the progress in performance of the teenagers is accompanied by lower brain volume.

Yet that progress in performance can lead to volume increase is in line with various studies, demonstrating that acquisition of a skill is accompanied by increase in gray brain matter (26, 65). Krafnick and colleagues (38) described that behavioral training effects in children with dyslexia are accompanied by rising brain volumes in the left anterior fusiform gyrus/hippocampus, left precuneus, right hippocampus and right anterior cerebellum. After this volume increase, a developmental effect similar to the neurotypical readers is occurring in the form of a decrease of local brain volume accompanied by an increase in the performance level. As these developments can be observed in regions associated to the reading network, this strongly suggests that preexisting structural differences in children are varied by experience-dependent structural change involving dendritic and synaptic pruning (13). The interaction effect can therefore be described as neuronal developmental delay, leading to partial compensation by a subsequent volume increase and then decrease with age and rising performance skills.

That volume changes do not proceed in a straightforward single direction (only lower brain volumes accompany better performance) is demonstrated by Linkersdörfer and colleagues (13): they describe that both, volume in- as well as decrease is associated with progress in performance. The results of the present study are thus further evidence for the flexibility of the human brain.

In sum, these morphometric and behavioral data combined appear to indicate that the differences in brain matter volume in the children are an early neuroanatomical signature of the dyslexia-specific reading problems. Additionally, even though the patients with dyslexia show progress in performance, it appears that the neuronal processes leading to this increase in performance are different from those in the neurotypical readers. Whereas neurotypical readers display a steady fine-tuning of the neural reading network and a decrease of gray matter, in readers with dyslexia progress in performance does not show these linear correlations.

### Methodological Issues

VBM is primarily a tool to investigate effects in gray matter, whereas its sensitivity in detecting effects in white matter is inferior because in *T1*-weighted MR images, this tissue type is characterized by the presence of large homogeneous regions with only small changes in signal. Changes in white matter fiber tracts can be better detected using Diffusion Tensor Imaging (DTI). However, this does not mean that white matter analysis is less reliable, but it is more difficult to detect effects compared to gray matter. Sophisticated methods such as DTI are simply more powerful at detecting effects in white matter, but when effects are found with VBM, they are no less reliable.

Because our findings are restricted to effects in the children only, one might ask whether our results are exaggerated due to normalizing them to a template for an older age group. Therefore, we additionally preprocessed and reanalyzed our children's data by creating a children-specific template. By using this alternative approach, we observe an almost identical pattern in the differences between the neurotypical reading children and children with dyslexia. Thus, it is very unlikely that the effects in children are driven by the use of a template, which is created from the whole sample of children, teenagers, and adults.

With respect to the bilateral findings across the brain, one might speculate that these findings could be caused by the fact that dyslexic children have smaller brains. However, we account for individual differences in brain size in our approach. Although segmented images are scaled by the extent of contraction or expansion due to spatial normalization, we do not scale by the linear effects of spatial normalization. Thus, overall brain size differences are corrected for, while local differences in the brains are preserved.

### Limitations of the Study

The drawback of our design is that it cannot uncover whether differences in brain volume are preexisting before reading acquisition and thus cause differences in reading skill development, or whether these anatomical differences result from the failure of learning to read. Additionally the cross-sectional design does not allow answering developmental questions in a straightforward manner. However as Casey and colleagues point out (60), longitudinal MRI studies investigating the structural brain development in children and teenagers (66, 67) observe similar patterns as cross-sectional studies (68–70). Thus, also cross-sectional investigations provide valuable information.

## Conclusion

Brain development is a lifelong process with regressive as well as progressive learning and experience based neuronal changes (26, 60). Longitudinal studies provide reliable evidence for a causal relationship between the learning experience and subsequent changes in brain volume. Looking at the current state of the literature, a straightforward conclusion for neuronal differences in brain volume between neurotypical normal and dyslexic readers is compounded due to the heterogeneity of VBM study results. This study attempts to shed light on these questions by maintaining the constancy of certain crucial experimental settings such as the diagnostic criteria, the methods of data measurement and data analysis while comparing different age groups of neurotypical and dyslexic readers. The VBM and behavioral data point out that reading deficits in individuals with dyslexia are associated with gray matter volume differences in the reading network compared to neurotypical readers and that in those two groups behavioral improvement in reading skills is reflected in different neuroanatomical patterns. Even though there is some compensation by an increase in the brain volume and subsequent network shaping, this neural baseline is not sufficient to allow for the development of neurotypical reading skills even in dyslexic adults.

Thus, this study, in its investigation of neuroanatomical and behavioral data over a wide age range is further evidence that dyslexia is a disorder with lifelong consequences, which is why consistent support for affected individuals in their educational and professional careers is of great importance. Longitudinal studies, which include investigations of the individually developing dyslexic brain as well as the question whether longitudinally (until well into adulthood) remediation after reading intervention leads to anatomical profiles similar to those of neurotypical readers are needed in order to come to understand whether these findings hold as a valid pattern or whether there is evidence of greater interindividual variance in the neuroanatomy of dyslexia.

## Data Availability Statement

The raw data supporting the conclusions of this article will be made available by the authors, without undue reservation.

## Ethics Statement

The studies involving human participants were reviewed and approved by Local Ethics Committee at the Jena University Hospital. Written informed consent to participate in this study was provided by the participants' legal guardian/next of kin.

## Author Contributions

CL and ML contributed to patients' recruitment and data collection. All authors contributed to data processing, participated in drawing up the manuscript, involved in the intellectual workup for the article, read, and approved the final manuscript.

## Funding

This work was supported by the [Deutsche Forschungsgemeinschaft] under Grant [LI 2659/2-1], the [Deutsche Forschungsgemeinschaft] under Grant [BL 435/3-1 and BL 435/3-2].

## Conflict of Interest

The authors declare that the research was conducted in the absence of any commercial or financial relationships that could be construed as a potential conflict of interest.

## Publisher's Note

All claims expressed in this article are solely those of the authors and do not necessarily represent those of their affiliated organizations, or those of the publisher, the editors and the reviewers. Any product that may be evaluated in this article, or claim that may be made by its manufacturer, is not guaranteed or endorsed by the publisher.
